# A prospective study on the use of ultralow-dose computed tomography with iterative reconstruction for the follow-up of patients liver and renal abscess

**DOI:** 10.1371/journal.pone.0246532

**Published:** 2021-02-12

**Authors:** Nieun Seo, Mi-Suk Park, Jun Yong Choi, Joon-Sup Yeom, Myeong-Jin Kim, Yong Eun Chung, Nam Su Ku

**Affiliations:** 1 Department of Radiology, Yonsei University College of Medicine, Seoul, Republic of Korea; 2 Department of Internal Medicine, Yonsei University College of Medicine, Seoul, Republic of Korea; 3 AIDS Research Institute, Yonsei University College of Medicine, Seoul, Republic of Korea; Humanitas Clinical and Research Center - IRRCS, ITALY

## Abstract

**Background:**

Radiation dose reduction is a major concern in patients who undergo computed tomography (CT) to follow liver and renal abscess.

**Objectives:**

The purpose of this study is to investigate the feasibility of ultralow-dose CT with iterative reconstruction (IR) to follow patients with liver and renal abscess.

**Methods:**

This prospective study included 18 patients who underwent ultralow-dose CT with IR to follow abscesses (liver abscesses in 10 patients and renal abscesses in 8 patients; ULD group). The control group consisted of 14 patients who underwent follow-up standard-dose CT for liver abscesses during the same period. The objective image noise was evaluated by measuring standard deviation (SD) in the liver and subcutaneous fat to select a specific IR for qualitative analysis. Two radiologists independently evaluated subjective image quality, noise, and diagnostic confidence to evaluate abscess using a five-point Likert scale. Qualitative parameters were compared between the ULD and control groups with the Mann-Whitney U test.

**Results:**

The mean CT dose index volume and dose length product of standard-dose CT were 8.7 ± 1.8 mGy and 555.8 ± 142.8 mGy·cm, respectively. Mean dose reduction of ultralow-dose CT was 71.8% compared to standard-dose CT. After measuring SDs, iDose level 5, which showed similar SD to standard-dose CT in both the subcutaneous fat and liver (*P* = 0.076, and *P* = 0.124), was selected for qualitative analysis. Ultralow-dose CT showed slightly worse subjective image quality (*P <* 0.001 for reader 1, and *P* = 0.005 for reader 2) and noise (*P* = 0.004 for reader 1, and *P* = 0.001 for reader 2) than standard-dose CT. However, the diagnostic confidence of ultralow-dose CT for evaluating abscess was comparably excellent to standard-dose CT (*P* = 0.808 for reader 1, and *P* = 0.301 for reader 2).

**Conclusions:**

Ultralow-dose CT with IR can be used in the follow-up of liver and renal abscess with comparable diagnostic confidence.

## Introduction

Intra-abdominal abscesses can be clinically suspected in patients with acute abdominal pain and fever. Appropriate diagnosis and management of intra-abdominal abscess is important, because mortality rates can be as high as 80 to 100% when abscesses are left untreated [1–3]. Even after initial percutaneous drainage, abscess recurrence can occur to increase morbidity and mortality rates [4–7]. Therefore, in those patients with intra-abdominal abscess, imaging studies for initial diagnosis and follow-up are essential. Contrast-enhanced computed tomography (CT) has been considered the best imaging modality for the accurate diagnosis of intra-abdominal abscess [8, 9]. CT also plays an important role when deciding treatment options and guiding the drainage procedure for abscess [10–12]. After appropriate treatment such as antibiotics or drainage procedures, most patients undergo follow-up CT scans to evaluate treatment response within a short time interval. According to a recently published study that included patients with inflammatory bowel disease and intra-abdominal abscess, the mean total number of follow-up CT performed during an abscess episode was 5.5 ± 3.7 in patients with total radiation dose > 50 mSv, and 2.4 ± 1.4 in those with total radiation dose < 50 mSv. The mean effective dose per follow-up CT was 5.96 ± 4.4 mSv [13]. Repeating CT scans to follow intra-abdominal abscesses inevitably increases a patient’s radiation exposure.

When following intra-abdominal abscesses such as liver and renal abscesses, CT with radiation doses lower than initial CT may be acceptable, because a diagnosis has already been established with the initial standard-dose CT. According to the “as low as reasonably achievable” principle, it is of utmost importance to reduce the radiation dose of CT while preserving diagnostic image quality [14]. Iterative reconstruction (IR) algorithms have widely been used to reduce image noise while maintaining image quality, which has enabled significant dose reduction of body CT [15–18].

Recently, a few studies have investigated the performance of ultralow-dose CT (with approximately 70–90% dose reduction compared to conventional CT) with various IR techniques in the abdomen, to see its potential for CT colonography, evaluation of urolithiasis, detection and characterization of focal liver lesions, or follow-up of patients with malignant lymphoma [19–22]. Furthermore, reducing radiation exposure is particularly important for benign diseases such as abdominal abscess for which we can expect normal life expectancy if a patient recovers without complications. In this study, we aimed to investigate the feasibility of using ultralow-dose abdominal CT with an IR algorithm to follow patients with liver and renal abscesses.

## Materials and methods

### Phantom study

We performed a phantom study using an anthropomorphic phantom (Model 701-G-ATOM Adult Male Phantom, CIRS, Norfolk, Virginia, USA) to determine the optimal radiation dose for ultralow-dose CT. The phantom was imaged on a CT device (Brilliance iCT, Philips Healthcare, Cleveland, OH) with the same parameters to be used in the clinical study other than the dose right index (DRI). DRI was developed to preserve consistent image quality regardless of estimated body weight. As routine clinical abdominopelvic CT scans are performed using DRI 14–16 in our institution, we aimed for at least 50–70% reduction in radiation dose compared to standard-dose CT when evaluating possible radiation doses for ultralow-dose CT. The radiation doses for each scan of the phantom with different DRI are presented in Table 1. Effective dose decreased 50.2% on the scan with DRI 9 compared with the scan with DRI 15. Based on the results of this phantom study, we chose DRI 9 for ultralow-dose CT in our clinical study.

**Table 1:** Radiation doses in the phantom experiment.

DRI	CTDIvol (mGy)	DLP (mGy·cm)	ED (mSv)
9	2.9	164.0	2.5
10	3.3	186.7	2.8
11	3.7	209.3	3.1
12	4.1	231.9	3.5
13	4.6	260.2	3.9
14	5.1	288.5	4.3
15	5.8	328.1	4.9

DRI, dose right index; CTDIvol, CT dose index volume; DLP, dose length product; ED, effective dose.

### Patients

This prospective study was approved by Institutional Review Board of Severance Hospital, Yonsei University Health System (No. 4-2015-0413), and written informed consent was obtained from all patients. From November 2015 to March 2019, patients who met the following criteria were enrolled: (a) patients who had clinically suspected intra-abdominal abscess, (b) patients who underwent initial standard-dose contrast-enhanced abdominopelvic CT in the emergency room, (c) and patients who agreed to participate in this study. Three to 6 weeks after the first CT scan, patients included in this study underwent ultralow-dose CT in a CT room in the Cancer Center. Among 20 patients who satisfied the above criteria, two patients were excluded whose raw CT data had been lost. Therefore, a total of 18 patients were included in the ultralow-dose (ULD) CT group (male:female, 7:11; mean age, 57.5 ± 13.6 years). For the controls, we retrospectively searched electronic medical records for patients with the same inclusion criteria but who underwent follow-up CT with the routine standard dose protocol in the same CT room during the same study period. Finally, 14 patients (male:female, 11:3; mean age, 64.8 ± 8.5 years) were included in the control group. In the ULD group, 10 patients had liver abscesses and 8 had renal abscesses, whereas all patients in the control group had liver abscesses.

### CT acquisition

All CT examinations in the study population included the portal venous phase (PVP), while the precontrast or late arterial phase was optionally obtained at the discretion of the performing physician. Radiation doses were estimated and images were analyzed on the PVP. Contrast-enhanced CT was performed after an intravenous injection of 2.0 mL/kg (up to a maximum 150 mL when patients weighed more than 75 kg) of iodinated contrast media (300 mgI/mL) followed by a bolus injection of 20 mL of saline chaser. Using the bolus tracking technique, CT scans in the PVP were obtained approximately 55 s after the attenuation of the abdominal aorta increased 100 HU compared to the baseline.

Initial CT scans in the emergency room were performed with a 128-detector CT (Revolution EVO, GE Healthcare, Waukesha, WI). Parameters for the CT scan were as follows: tube voltage, 120 kV; tube current, 100–350 mAs with tube current modulation; noise index, 22.52; pitch, 0.984; rotation time, 0.5 s; beam collimation, 40 mm, and section thickness, 3 mm. The protocol for the initial CT scan in the emergency room was identical between the ULD and control groups.

Follow-up CT was performed with a 128 slice CT with double z-sampling (Brilliance iCT, Philips Healthcare, Cleveland, OH). In this CT machine, the DRI was used to adjust radiation exposure according to patient group. By using the DRI, the default mAs was determined based on estimated body weight. For the control group, DRI was 15 according to the routine CT protocol in our institution. For ultralow-dose CT (the ULD group), DRI was set as 9 in the PVP to reduce radiation dose 50–70% compared to standard-dose CT based on the previous phantom experiment. After determining the default mAs with DRI, AEC was also applied. CT parameters except DRI were the same between the ULD and control groups as follows: tube voltage, 120 kV; pitch, 0.899; rotation time, 0.5 s; beam collimation, 128 × 0.625 mm, and section thickness, 3 mm. To measure the radiation dose, the CT dose index volume (CTDIvol) and dose length product (DLP) in the PVP were recorded.

### Image reconstruction

In both the ULD and control groups, initial standard-dose CT in the emergency room was reconstructed using the 20% Adaptive Statistical Iterative Reconstruction (ASIR) algorithm which was incorporated in the routine protocol of our institution. For follow-up low-dose CT in the ULD group, two IR methods, iDose and iterative model reconstruction (IMR), were applied in this study. iDose is a hybrid IR algorithm, with different levels (1 to 6 or 7 depending on the scan protocol) of noise reduction according to the degree of noise removal. A higher iDose level indicates a higher strength of noise removal, with an increase of approximately 11%–55% compared to the corresponding filtered back projection (FBP) reconstruction [23]. IMR is an advanced model- and knowledge-based IR algorithm [24]. Similar to iDose, image noise is adjusted to IMR levels (1–3), and higher IMR levels indicate a larger degree of noise reduction. Ultralow-dose CT images during the PVP in the ULD group were reconstructed using FBP, iDose and IMR in the console. Therefore, nine imaging sets (iDose levels 1–6, and IMR levels 1–3) were generated for IR in the ULD group. The reconstructed slice thickness was 3 mm without overlap for all reconstruction image sets. Follow-up standard-dose CT in the control group was reconstructed with only iDose level 3 according to the routine protocol of our institution.

### Image analysis

Quantitative analysis was performed in the ULD group to select a reconstruction phase of the follow-up ultralow-dose CT which showed the most similar objective image noise with initial standard-dose CT. Then, this reconstruction phase of ultralow-dose CT was used for further qualitative image analysis. One abdominal radiologist (with 7 years of experience in abdominal CT) performed the quantitative measurements using a picture archiving and communication system (Centricity, GE Healthcare). Objective image noise was determined by the standard deviation (SD) of the CT number within a region of interest (ROI) in the subcutaneous fat and liver, respectively. In the subcutaneous fat, three circular ROIs (85.7–135.5 mm^2^) were drawn as large as possible in different levels of the abdominal wall while avoiding vessels or heterogeneous areas. In the liver, three circular ROIs (94.5–156.8 mm^2^) were also drawn as large as possible in different locations of the liver (right anterior section, right posterior section, and left hemiliver). These ROIs were placed carefully to exclude blood vessels, bile duct, and focal hepatic lesions. The contrast-to-noise ratio (CNR) was measured as (MD_lesion_—MD_perilesion_)/SD_fat_. MD_lesion_ was the mean lesion density (abscess), MD_perilesion_ was the mean density of the adjacent liver (or renal cortex), and SD_fat_ was the SD measured in the subcutaneous fat. The location and size of the measured ROIs were identical between the initial standard-dose CT scan and the follow-up ultralow-dose CT scan. The mean values of the three ROIs were used for further analysis.

For qualitative analysis, two board-certified radiologists with 7 and 13 years of experience in abdominal CT independently reviewed the CT images. Prior to image analysis, imaging data were anonymized to exclude patient information, thus both reviewers were blinded to the patient group (ULD or control group). For both the ULD and control groups, the image quality of the follow-up CT scans was compared with the initial CT scans. Because follow-up CT in the ULD group had many reconstructed phases (FBP and nine IR imaging sets), one reconstruction phase was selected based on the aforementioned quantitative analysis. Overall image quality, subjective image noise, artificial texture, and diagnostic confidence for the evaluation of abscess were evaluated using a five-point Likert scale, and higher scores indicated better image quality. For overall image quality, scores were defined as follows: 1, much poorer image quality than initial CT; 2, slightly poorer than initial CT; 3, similar to the initial CT; 4, slightly better than initial CT; and 5, much better than initial CT. For subjective noise and artificial texture, scores were graded as follows: 1, much more noise (artificial texture) than initial CT; 2, slightly more than initial CT; 3, similar to initial CT; 4, slightly less than initial CT, and 5, much less than initial CT. Diagnostic confidence for the evaluation of abscess was scored using the following criteria: 1, non-diagnostic; 2, considerably degrading diagnostic confidence; 3, slightly degrading diagnostic confidence; 4, minimally degrading diagnostic confidence; and 5, not affecting diagnostic confidence.

### Statistical analysis

Baseline patient demographics and radiation dose were compared between the ULD group and control group using independent *t*-tests for continuous variables and Pearson’s chi-squared test or Fisher’s exact test for categorical variables. In the ULD group, SDs measured in the subcutaneous fat and liver, and CNRs were compared between the initial CT and each reconstruction phase of the follow-up CT using paired *t*-tests. To compare qualitative parameters between the ULD and control groups, and between patients with body mass index (BMI) < 25 kg/m^2^ and patients with ≥ 25 kg/m^2^, the Mann-Whitney U test was used. Interobserver agreement for qualitative analysis was evaluated between the two reviewers by percent agreement and prevalence-adjusted and bias-adjusted kappa (PABAK) [25]. PABAK statistics were interpreted as follows: 0.01–0.20, slight agreement; 0.21–0.40, fair agreement; 0.41–0.60, moderate agreement; 0.61–0.80, substantial agreement; and 0.81–0.99, almost perfect agreement [26]. Statistical analyses were conducted using SPSS Statistics version 25.0 (IBM Corp., New York, NY). *P* values smaller than 0.05 were considered statistically significant.

## Results

### Patient demographics and radiation dose measurements

The ULD group consisted of 18 patients, and the control group 14 patients. Patient demographics and CT radiation doses are summarized in Table 2. BMI was not significantly different between the ULD and control groups (23.4 ± 3.2 vs. 22.9 ± 3.4, *P* = 0.667). The size of the abscess measured on follow-up CT was not significantly different between the ULD and control groups (1.7 ± 0.3 cm vs. 1.8 ± 1.5 cm, *P* = 0.807). Intervals between the initial and follow-up CT were comparable between the ULD and control groups (36.9 ± 8.1 vs. 33.8 ± 10.9 days, *P* = 0.338). Regarding radiation dose, the CTDIvol and DLP of the initial CT were not significantly different between the ULD and control groups (9.2 ± 3.5 vs. 9.7 ± 2.8 mGy, *P* = 0.536 for CTDIvol; and 521.9 ± 229.5 vs. 561.6 ± 182.6 mGy·cm, *P* = 0.398 for DLP). The CTDIvol and DLP of the follow-up CT were significantly lower in the ULD group compared to the control group (2.4 ± 0.6 vs. 8.7 ± 1.8 mGy, *P* < 0.001 for CTDIvol; and 151.5 ± 42.5 vs. 555.8 ± 142.8 mGy·cm, *P* < 0.001 for DLP). Compared to the initial standard-dose CT in the ULD group, ultralow-dose CT showed a mean dose reduction of 71.8% (median, 72.8%; range, 57.0–80.3%) for CTDIvol and 68.4% (median, 71.0%; range, 49.2–76.8%) for DLP, respectively.

**Table 2:** Patient demographics and radiation dose.

	ULD group (n = 18)	Control group (n = 14)	*P* value
Age (Years)	57.5 ± 13.6	64.8 ± 8.5	0.152
Sex			0.059
Male	7 (38.9%)	11 (78.6%)	
Female	11 (61.1%)	3 (21.4%)	
BMI (kg/m^2^)	23.4 ± 3.2	22.9 ± 3.4	0.667
<18.5	0	2 (14.3%)	
18.5–24.9	14 (77.8%)	7 (50%)	
≥25	4 (22.2%)	5 (35.7%)	
Diagnosis			0.034
Liver abscess	10 (55.6%)	14 (100%)	
Kidney abscess	8 (44.4%)	0 (0%)	
Size of abscess (cm)			
On initial CT	3.1 ± 2.0	2.6 ± 1.5	0.457
On follow-up CT	1.7 ± 0.3	1.8 ± 1.5	0.807
CT interval (days)*	36.9 ± 8.1	33.8 ± 10.9	0.338
Radiation dose			
CTDIvol of initial CT (mGy)	9.2 ± 3.5	9.7 ± 2.8	0.536
CTDIvol of follow-up CT (mGy)	2.4 ± 0.6	8.7 ± 1.8	<0.001
DLP of initial CT (mGy·cm)	521.9 ± 229.5	561.6 ± 182.6	0.398
DLP of follow-up CT (mGy·cm)	151.5 ± 42.5	555.8 ± 142.8	<0.001

Data are mean ± standard deviation.

*Time interval between initial and follow-up CT scans.

ULD, ultralow-dose; BMI, body mass index; CTDIvol, CT dose index volume; DLP, dose length product.

### Quantitative analysis

The objective image noises of initial and follow-up CT scans in the ULD group are summarized in Table 3. In the subcutaneous fat, the mean SD of the initial CT was 11.6 ± 1.1 HU. The SDs measured on iDose 4 (11.5 ± 1.9, *P* = 0.932) and iDose 5 (10.7 ± 1.7, *P* = 0.076) of follow-up ultralow-dose CT were comparable to initial CT. In the liver, the mean SD of the initial CT was 14.7 ± 2.9 HU. On follow-up CT, SDs measured on iDose 5 (16.2 ± 2.8, *P* = 0.124) and iDose 6 (13.9 ± 2.2, *P* = 0.329) were not significantly different from those measured on initial CT. Thus, iDose 5 was selected for further qualitative image analysis because it showed similar SDs in both the subcutaneous fat and liver. Regarding CNR, initial standard-dose CT and follow-up CT scans with iDose showed similar CNR values in the ULD group, and the CNR was even higher with IMR, probably due to decreases in image noise.

**Table 3:** Objective image noise (SD) of initial and follow-up CT scans in the ULD group.

	Initial CT	Follow-up ultralow-dose CT									
	Standard	FBP	iDose 1	iDose 2	iDose 3	iDose 4	iDose 5	iDose 6	IMR 1	IMR 2	IMR 3
SQ fat	11.6 ± 1.1	22.0 ± 4.7	14.4 ± 2.4	13.4 ± 2.4	12.8 ± 2.2	11.5 ± 1.9	10.7 ± 1.7	9.7 ± 1.7	7.8 ± 1.1	6.8 ± 1.0	5.7 ± 0.9
*P*-value		< 0.001	< 0.001	0.010	0.035	0.932	0.076	0.001	< 0.001	< 0.001	< 0.001
Liver	14.7 ± 2.9	39.9 ± 10.1	23.0 ± 3.4	21.5 ± 3.1	20.3 ± 3.2	18.3 ± 2.7	16.2 ± 2.8	13.9 ± 2.2	9.8 ± 1.4	8.0 ± 1.2	6.1 ± 1.1
*P*-value		< 0.001	< 0.001	< 0.001	< 0.001	0.001	0.124	0.329	< 0.001	< 0.001	< 0.001
CNR	7.4 ± 2.8	5.1 ± 3.7	7.3 ± 3.8	8.0 ± 4.3	8.4 ± 4.3	8.9 ± 5.0	9.5 ± 4.9	10.2 ± 4.6	14.1 ± 8.6	16.0 ± 11.1	19.1 ± 12.1
*P*-value		0.028	0.802	0.730	0.300	0.397	0.090	0.060	0.006	0.002	0.001

Data are mean ± standard deviation.

*P*-values are for differences between standard-dose initial CT and each reconstruction of follow-up ultralow-dose CT.

SD, standard deviation; ULD, ultralow-dose; SQ, subcutaneous; CNR, contrast-to-noise ratio; FBP, filtered back projection; IMR, iterative model reconstruction.

### Qualitative analysis

**Fig 1:**
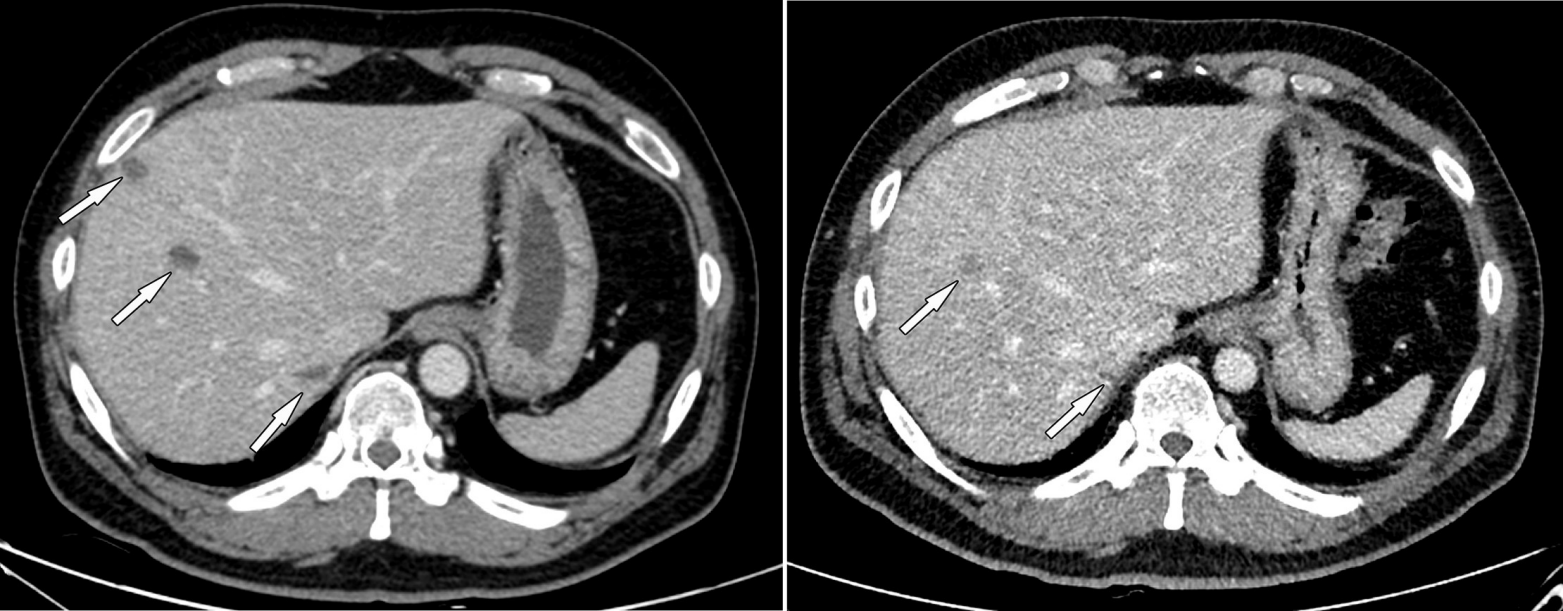
Contrast-enhanced CT images of a 46-year-old man with liver abscesses. Body mass index was 30.7 kg/m^2^. (A) Initial standard-dose CT shows several low attenuation lesions in the right hemiliver (arrows), suggestive of liver abscesses. (B) Follow-up ultralow-dose CT shows the liver abscesses decrease in size (arrows). The CT dose index volume of initial CT and follow-up CT was 15.4 mGy and 3.7 mGy, respectively. Image quality and noise were worse on follow-up CT, but diagnostic confidence was relatively preserved. The scores for image quality, noise, and diagnostic confidence were 2, 1, and 5 for reader 1, and 1, 1, and 4 for reader 2.

**Fig 2:**
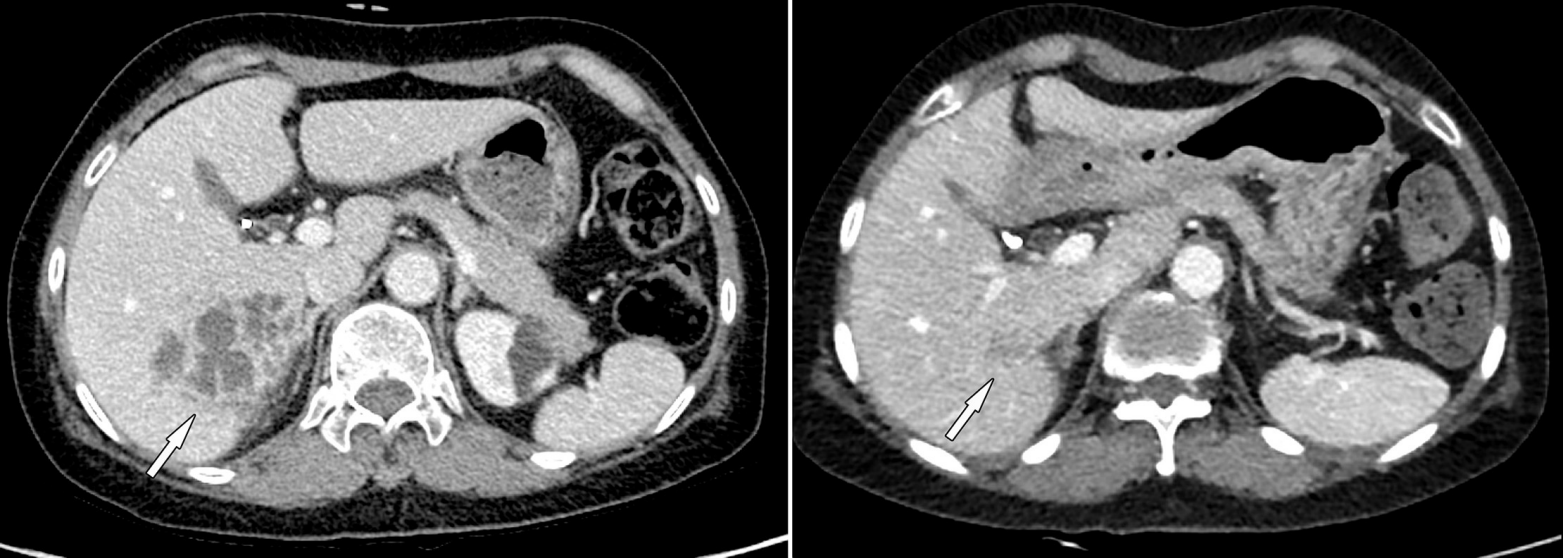
Contrast-enhanced CT images of a 61-year-old woman with liver abscesses. Body mass index was 20.4 kg/m^2^. (A) Initial standard-dose CT shows clustered peripheral enhancing lesions in the right posterior liver (arrow), suggestive of liver abscesses. (B) Follow-up ultralow-dose CT shows liver abscess nearly resolved (arrow). The CT dose index volume of initial CT and follow-up CT was 5.7 mGy and 1.8 mGy, respectively. Image quality and noise were comparable between initial CT and follow-up CT, and the diagnostic confidence of follow-up ultralow-dose CT was excellent. The scores for image quality, noise, and diagnostic confidence were 3, 3, and 5 for both readers.

Overall image quality (2.3 ± 0.5 vs. 3.1 ± 0.5, *P* < 0.001 for reader 1, and 2.3 ± 0.7 vs. 3.1 ± 0.7, *P* = 0.005) and subjective image noise (2.1 ± 0.8 vs. 2.9 ± 0.6, *P* = 0.004 for reader 1, and 2.2 ± 0.6 vs. 3.2 ± 0.7, *P* = 0.001 for reader 2) of follow-up CT in the ULD group were significantly worse than the control group (Table 4). Artificial texture of follow-up CT was not significantly different between the ULD and control group (*P* = 750 for reader 1, and *P* = 0.694 for reader 2). Despite slightly worse image quality and noise in the ULD group, the diagnostic confidence of follow-up ultralow-dose CT for the evaluation of abscess (4.9 ± 0.3 vs. 5.0 ± 0.0, *P* = 0.808 for reader 1, and 4.7 ± 0.6 vs. 5.0 ± 0.0, *P* = 0.301 for reader 2) was comparable to standard-dose CT (Figs 1 and 2). For abscess size, there was no significant difference in the diagnostic confidence in the control group (5.0 ± 0.0 vs. 5.0 ± 0.0, *P* > 0.999 for both readers). Also in the ULD group, there was no significant difference in the diagnostic confidence (4.9 ± 0.3 vs. 5.0 ± 0.0, *P* = 0.791 for reader 1; and 4.6 ± 0.7 vs. 4.9 ± 0.4, *P* = 0.659 for reader 2) according to abscess size (< 2 cm vs. ≥ 2 cm). All of these qualitative parameters did not significantly differ between patients with BMI < 25 kg/m^2^ and those with BMI ≥ 25 kg/m^2^ in both the ULD and control groups (S1 Table).

**Table 4:** Qualitative analysis.

	ULD group (n = 18)	Control group (n = 14)	*P* value
Overall image quality			
Reader 1	2.3 ± 0.5	3.1 ± 0.5	<0.001
Reader 2	2.3 ± 0.7	3.1 ± 0.7	0.005
Noise			
Reader 1	2.1 ± 0.8	2.9 ± 0.6	0.004
Reader 2	2.2 ± 0.6	3.2 ± 0.7	0.001
Artificial texture			
Reader 1	3.0 ± 0.0	3.1 ± 0.3	0.750
Reader 2	3.2 ± 0.5	3.1 ± 0.5	0.694
Diagnostic confidence			
Reader 1	4.9 ± 0.3	5.0 ± 0.0	0.808
Reader 2	4.7 ± 0.6	5.0 ± 0.0	0.301

Data are mean ± standard deviation.

ULD, ultralow-dose.

Interobserver agreement for qualitative analysis was moderate for overall image quality (PABAK, 0.531) and noise (0.453), and substantial to almost perfect for artificial texture (0.648) and diagnostic confidence on abscess evaluation (0.883, Table 5).

**Table 5:** Interobserver agreement for qualitative analysis.

	% agreement	PABAK	95% CI
Overall image quality	59.4	0.531	0.393, 0.670
Noise	50.0	0.453	0.315, 0.592
Artificial texture	71.9	0.648	0.510, 0.787
Diagnostic confidence	90.6	0.883	0.744, 1.021

PABAK, Prevalence-Adjusted and Bias-Adjusted Kappa; CI, confidence interval.

## Discussion

This study investigated the feasibility of using ultra-low dose CT to follow intra-abdominal abscesses in adult patients. Using our protocol, the radiation dose of ultralow-dose CT (mean CTDIvol of 2.4 mSv) was approximately 70% lower than standard-dose CT. The subjective image quality and noise of follow-up ultralow-dose CT with IR were slightly worse than standard-dose CT in the control group. However, ultralow-dose CT and standard-dose CT did not differ in the diagnostic confidence to evaluate treatment response and to determine management plans. Also, when patients were classified according to BMI, all these qualitative parameters (overall image quality, noise, artificial texture, and diagnostic confidence) did not differ between patients with BMI < 25 kg/m^2^ and those with BMI ≥ 25 kg/m^2^ in both the ULD and control groups.

In patients with suspected intra-abdominal abscess such as liver or renal abscess, contrast-enhanced CT is usually required to establish a diagnosis, and to evaluate the size, anatomy, and characteristics of the abscess [27, 28]. After appropriate management, patients usually undergo follow-up CT when clinical symptoms persist or when treatment response needs to be evaluated. A recent study of patients with inflammatory bowel disease reported the mean total effective dose as 51.6 ± 44.7 mSv during an abscess episode, although this included fluoroscopic studies for drainage procedures as well as intervening CT scans [13]. In contrast to initial CT performed to establish a diagnosis, the not perfect image quality of follow-up CT for intra-abdominal abscess can be tolerated, because the primary disease has already been diagnosed and located on initial CT. One study showed that ultralow-dose CT that reduced radiation dose by 72% compared to conventional CT could detect intra-abdominal abscess in all patients with Crohn’s disease (3/3) [29]. Our study which included a larger number of patients with hepatic and renal abscesses also confirmed this hypothesis, although further studies with more patients with different types of abscesses or other ethnicities are warranted.

Among the two IR algorithms available in this study, iDose and IMR, iDose level 5 was selected for the ultralow-dose CT protocol, based on measurements of objective image noise. These two IR algorithms use different mechanisms for noise reduction: iDose is an IR algorithm that works in the combined projection and image domains, whereas IMR is a model-based IR algorithm [30–32]. The most essential difference between iDose and IMR is that IMR decreases both quantum noise and non-random noise intrinsic to the geometry and optics of the imaging system [33, 34]. According to previous studies, IMR can provide more dramatic dose reduction, but obtains more blotchy images and poor demarcation of anatomic structures which deteriorates its diagnostic confidence [34, 35]. Therefore, several previous studies have recommended the use of an intermediate level of iDose (3–4) to preserve the image quality of abdominopelvic CT [34, 36]. Based on our study results, we suggest that a relatively higher IR level (iDose level 5) could be used with ultralow-dose CT to follow liver or renal abscess, while allowing more dramatic dose reduction.

This study has several limitations. First, the small number of patients included in our analysis may limit the power of this study. Also, the BMI of our study population was quite low (mean ± SD, 23.4 ± 3.2), probably because it was made up of Asian patients. Inclusion of more obese patients could possibly decrease the diagnostic ability of ultralow-dose CT. Second, the different exam dates of the initial and follow-up CT scans may limit the side-by-side comparison of qualitative image parameters and abscess. However, the main focus of this study was to evaluate the feasibility of using ultralow-dose CT to follow liver and renal abscess. Furthermore, repeated acquisition of CT scans at the same time is unethical due to increased radiation exposure. Third, we compared different patient groups with different pathologies between the ULD and control groups. ULD group consisted of both liver or kidney abscesses, whereas the control group included patients with liver abscess only. Therefore, we were not able to compare other associated findings such as thrombophlebitis in the portal vein between the study and control groups. Finally, specific IR algorithms from a CT scanner from only one vendor were used. Also, only a specific IR method (iDose 3) was available for standard-dose CT compared to the 10 reconstructed datasets of ultralow-dose CT, because standard-dose CT was performed according to routine CT protocol. Hence, further studies that incorporate various IR techniques from other vendors are required to generalize our study results.

In conclusion, ultralow-dose CT combined with the IR technique can achieve approximately 70% dose reduction relative to conventional CT and can be used in the follow-up of liver and renal abscess with comparable diagnostic confidence.

## Supporting information

S1 Table Qualitative analysis according to body mass index. (DOCX)

## Abbreviations

CT: computed tomography
IR: iterative reconstruction
ULD: ultralow-dose
SD: standard deviation
PVP: portal venous phase
DRI: dose right index
AEC: automatic exposure control
CTDIvol: computed tomography dose index volume
ASIR: Adaptive Statistical Iterative Reconstruction
IMR: iterative model reconstruction
FBP: filtered back projection
ROI: region of interest
BMI: body mass index
