# Supplementary material for: A prospective study on the use of ultralow-dose computed tomography with iterative reconstruction for the follow-up of patients liver and renal abscess
Source: PLoS One. 2021 Feb 12;16(2):e0246532. doi: 10.1371/journal.pone.0246532 (PMC7880451; doi:10.1371/journal.pone.0246532)
Supplement: S1 Table — (DOCX) [file pone.0246532.s001.docx]

S1 Table. Qualitative analysis according to body mass index.

|  | Ultralow-dose group (n = 18) | | | Control group (n = 14) | | |
| --- | --- | --- | --- | --- | --- | --- |
|  | BMI < 25  (n = 14) | BMI ≥ 25  (n = 4) | *P* value | BMI < 25  (n = 9) | BMI ≥ 25  (n = 5) | *P* value |
| Overall image quality |  |  |  |  |  |  |
| Reader 1 | 2.4 ± 0.5 | 2.0 ± 0.0 | 0.327 | 3.1 ± 0.4 | 3.0 ± 0.7 | 0.606 |
| Reader 2 | 2.5 ± 0.7 | 1.8 ± 0.4 | 0.101 | 3.3 ± 0.5 | 3.2 ± 0.8 | 0.797 |
| Noise |  |  |  |  |  |  |
| Reader 1 | 2.3 ± 0.7 | 1.5 ± 0.5 | 0.101 | 3.0 ± 0.6 | 2.8 ± 0.8 | 0.606 |
| Reader 2 | 2.4 ± 0.6 | 1.8 ± 0.4 | 0.192 | 3.4 ± 0.5 | 3.2 ± 0.8 | >0.999 |
| Artificial texture |  |  |  |  |  |  |
| Reader 1 | 3.0 ± 0.0 | 3.0 ± 0.0 | >0.999 | 3.0 ± 0.0 | 3.2 ± 0.4 | 0.606 |
| Reader 2 | 3.1 ± 0.5 | 3.5 ± 0.5 | 0.277 | 3.0 ± 0.6 | 3.2 ± 0.4 | 0.606 |
| Diagnostic confidence |  |  |  |  |  |  |
| Reader 1 | 4.9 ± 0.3 | 5.0 ± 0.0 | 0.878 | 5.0 ± 0.0 | 5.0 ± 0.0 | >0.999 |
| Reader 2 | 4.9 ± 0.4 | 4.3 ± 0.8 | 0.277 | 5.0 ± 0.0 | 5.0 ± 0.0 | >0.999 |
| Data are mean ± standard deviation.  BMI, body mass index (unit: kg/m^2^) | | | | | | |
